# Endoscopic endonasal transcavernous posterior clinoidectomy with pituitary transposition for resection of a craniopharyngioma

**DOI:** 10.3171/2026.7.FOCVID2699

**Published:** 2026-10-01

**Authors:** Binbin Wang, Gang Cheng, Chao Tao

**Affiliations:** Department of Neurosurgery, The First Affiliated Hospital of Nanjing Medical University, Nanjing, Jiangsu Province, China

**Keywords:** craniopharyngioma, skull base, endoscopic endonasal surgery, surgical video

## Abstract

A 37-year-old male presented with diplopia for 3 weeks. MRI revealed a large solid suprasellar and retrosellar lesion highly suggestive of craniopharyngioma. Endoscopic endonasal transcavernous posterior clinoidectomy with pituitary transposition was performed. This approach provides sufficient access to the interpeduncular and prepontine cisterns, enabling gross-total resection. No new permanent neurological deficits were observed postoperatively.

The video can be found here: https://stream.cadmore.media/r10.3171/2026.7.FOCVID2699

## Transcript

This video demonstrates endoscopic endonasal transcavernous posterior clinoidectomy with pituitary transposition for resection of a craniopharyngioma.

### 0:33

The patient is a 37-year-old male who presented with a 3-week history of diplopia. Neurological examination revealed right abducens nerve palsy. Preoperative visual field testing and pituitary function were normal.

### 0:50

MRI revealed a large solid suprasellar and retrosellar lesion, and a CT scan demonstrated a prominent calcified component. The radiological diagnosis was consistent with craniopharyngioma.

### 1:06

Due to obstruction by the pituitary gland, only the suprasellar portion of the tumor could be visualized through the space between the optic chiasm inferiorly and the pituitary gland superiorly. Exposure of the retrosellar component required posterior clinoid process removal and pituitary transposition.

### 1:28

The endoscopic endonasal approach is performed. A right-sided vascularized nasoseptal flap is first elevated. Partial resection of the right middle turbinate and ethmoidectomy are performed to improve exposure, followed by posterior nasal septectomy. A reverse flap is then prepared. The contralateral nasal mucosa is transected and brought forward along the exposed right-sided cartilage.

### 1:58

A wide bilateral sphenoidotomy is completed. This endoscopic view shows the ventral skull base, with extensive sellar and parasellar exposure achieved. The bone overlying the face of the sella, anterior wall of the right cavernous sinus, and clival recess is carefully removed. The tuberculum sellae is drilled superiorly up to the sphenoid limbus. Full skeletonization of the right paraclinoidal internal carotid artery is completed. Once thinned with a high-speed drill, the bone is carefully peeled with a Kerrison rongeur and removed.

### 2:35

The anterior wall of the right cavernous sinus is opened, and we enter the cavernous sinus. Venous bleeding is controlled with intracavernous injection of Surgifoam (Ethicon, Inc.). The inferior hypophyseal artery is identified, coagulated, and transected. Since the bone overlying the anterior wall of the right cavernous sinus and the right upper clivus had been drilled in advance, the right posterior clinoid process was isolated. Pituitary transposition allows full exposure of the right posterior clinoid process and peels it free from its dural attachment. The medial wall of the cavernous sinus remains attached to the pituitary gland during its medial and superior mobilization.

### 3:22

The dura covering the prechiasmatic region is then opened. The proximal and distal rings as well as the diaphragma sellae are transected. Subsequently, the dura of the dorsum sellae and clivus is incised downward. By displacing the pituitary gland, we achieve full exposure of the tumor located in the interpeduncular and prepontine cisterns.

### 4:00

Piecemeal intracapsular debulking of the tumor is performed, including removal of its calcified component. As additional space is created through further debulking, the surrounding neural structures are carefully dissected away from the tumor capsule. The capsule is separated from the basilar artery, posterior cerebral artery, superior cerebellar artery, and oculomotor nerve.

### 4:37

We sharply dissected the tumor from the pituitary gland and pituitary stalk with scissors. This is the site of tumor origin.

### 4:53

The mammillary bodies and subsequently the basilar artery apex come into view. Dissecting the tumor away from the brainstem remains the most challenging step of the procedure. Preserving the arachnoid membranes is critical to protect the brainstem and its perforating arteries. Sufficient internal debulking must be performed before dissecting the tumor capsule. The safe plane of dissection is strictly between the tumor capsule and the brainstem arachnoid. Gross-total resection of the tumor is achieved.

### 5:35

Complete mobilization of the pituitary gland provides unobstructed surgical access to the entire interpeduncular and prepontine cisterns.

### 5:50

Skull base reconstruction is performed using a two-layer technique. A DuraGen inlay graft is placed to primarily close the dural defect. This is followed by placement of the vascularized nasoseptal flap, which is secured in position with Surgicel. Finally, pack the sphenoid sinus with iodoform gauze.

### 6:18

Postoperative MRI confirmed gross-total resection of the tumor. The patient developed transient diabetes insipidus, which resolved completely within 1 month. His right abducens nerve palsy improved within 1 week postoperatively and eventually resolved entirely. No other new neurological deficits were noted. A lumbar drain was placed for several days and then removed. No postoperative cerebrospinal fluid leak was encountered. Pituitary function remained normal postoperatively. The patient recovered well. The final pathological diagnosis was adamantinomatous craniopharyngioma.

### 7:02

References.^1–6^

## Disclosures

The authors report no conflict of interest concerning the materials or methods used in this study or the findings specified in this publication.

## Author Contributions

Primary surgeon: Tao. Assistant surgeons: Wang, Cheng. Editing and drafting the video and abstract: Tao. Critically revising the work: Tao, Cheng. Reviewed submitted version of the work: all authors. Approved the final version of the work on behalf of all authors: Tao. Supervision: Tao.

## Supplemental Information

### Patient Informed Consent

The necessary patient informed consent was obtained in this study.
